# The Colonization Dynamics of the Gut Microbiota in Tilapia Larvae

**DOI:** 10.1371/journal.pone.0103641

**Published:** 2014-07-29

**Authors:** Christos Giatsis, Detmer Sipkema, Hauke Smidt, Johan Verreth, Marc Verdegem

**Affiliations:** 1 Aquaculture and Fisheries Group, Wageningen University, Wageningen, The Netherlands; 2 Laboratory of Microbiology, Wageningen University, Wageningen, The Netherlands; Fish Vet Group, Thailand

## Abstract

The gut microbiota of fish larvae evolves fast towards a complex community. Both host and environment affect the development of the gut microbiota; however, the relative importance of both is poorly understood. Determining specific changes in gut microbial populations in response to a change in an environmental factor is very complicated. Interactions between factors are difficult to separate and any response could be masked due to high inter-individual variation even for individuals that share a common environment. In this study we characterized and quantified the spatio-temporal variation in the gut microbiota of tilapia larvae, reared in recirculating aquaculture systems (RAS) or active suspension tanks (AS). Our results showed that variation in gut microbiota between replicate tanks was not significantly higher than within tank variation, suggesting that there is no tank effect on water and gut microbiota. However, when individuals were reared in replicate RAS, gut microbiota differed significantly. The highest variation was observed between individuals reared in different types of system (RAS vs. AS). Our data suggest that under experimental conditions in which the roles of deterministic and stochastic factors have not been precisely determined, compositional replication of the microbial communities of an ecosystem is not predictable.

## Introduction

The gut of fish harbors a diverse microbial community. It provides niches for adherence, colonization and proliferation of mutualistic, benign commensal and pathogenic microbial species that affect many physiological and immunological functions of the host [1]–[3]. The microbial community in the gut changes with the developmental stage of the host and constantly adapts to the nutritional and environmental situation [4]–[7]. Impacts on fish gut microbiota are more pronounced during early ontogenetic stages when the fish gut is not yet fully developed and the immune system is immature [8].

However, due to high inter-individual variation between fish and rapid changes in the microbial community composition during early life stages, it is difficult to relate changes in gut microbiota to alterations of a single factor. It has been suggested that inter-individual variation in gut microbial community composition both in humans [9] and animals [10] might mask treatment effects. High individual variation was suggested as the reason for not detecting differences in gut microbiota in Atlantic salmon (*Salmo salar*) fed with different diets [11]. High inter-individual variation in quantity, diversity and richness of gut bacteria was also observed between individuals from the same tank in Bluefin tuna [12] as well as in cod larvae [13].

Inter-individual variation in gut microbiota between individuals reared under the same conditions can be partially explained by stochastic processes [14]. However, “stochastic variation” cannot just be considered as “noise”. To comprehend the full range of genetic and metabolic diversity of gut microbiota, it is very important to characterize and quantify the inter- and intra-individual variation in space and time. In fact, the characterization of the variation between identically reared individuals can serve as baseline to determine the contribution of stochastic factors to the overall variation.

In this study we characterized and quantified the spatio-temporal variation of water and gut microbiota of Nile tilapia larvae, reared for six weeks in two replicate recirculation aquaculture systems (RAS). The location effects on larvae gut microbiota were compared for individuals reared within the same or between replicate tanks, and between replicate RAS systems. To determine the generality of any pattern observed in the RAS, and to avoid any affinity of the results with the specific habitat (RAS), temporal and replication effects were also studied in replicate active suspension (AS) systems also known as zero-exchange activated sludge systems or biofloc systems [15].

## Materials and Methods

### Ethics statement

The experiment was approved by the Ethical Commission for Animal Experiments of Wageningen University (Project Name: Promicrobe; Registration code: 2009055d).

### Experimental animals and set up

Three to four days old fertilized Nile tilapia eggs, obtained from TilAqua International (Velden, the Netherlands) were incubated at 27°C. Two different culture systems were used to rear the newly hatched larvae: a recirculating aquaculture system (RAS) with two replicates (Ra and Rb), and an active suspension (AS) system with five replicates (AS 1-5). Each RAS contained five 20-L tanks and the tanks were connected to the same water purification unit. The two replicate RAS systems were not connected to each other. The five 120-L AS tanks were independent units and they did not share the same water. Initially, all systems were filled with water from the same source. In addition, before the start of the experiment, water and filter materials from the two RAS were mixed. Water of the five AS systems was treated similarly. The larvae were incubated together in a common tank before stocking. In each tank, 100 randomly selected swim-up larvae (7 days post fertilization) were introduced before the first feed application. Feeding started 9 days post fertilization (referred to as day 0; D00) and was continued for 42 days. Each day, larvae were fed with 0.5 mm commercial starter tilapia diet (F-0.5 GR Pro Aqua Brut – Trouw Nutrition, France) until apparent satiation for maximum 30 minutes at 9.00, 12.30 and 16.00 hours. The same type of feed, originating from a common batch, was used throughout the 42 day experimental period. Feed pellets were introduced slowly while observing feeding behaviour, and administration stopped when it took more than 15 seconds before fishes reacted to newly fed pellets. Just before the first feeding, water and gut samples were collected, to determine the “initial” microbiota. Other samples were taken before the first daily feeding on day 07, 14, 28 and 42 (Figure S1).

Water physicochemical characteristics were maintained at safe levels for Nile tilapia larvae (pH 6.6–8.5, temperature 26–28°C, NH_3_-N<0.2 mg l^−1^, NO_2_-N<1 mg l^−1^ and DO >5 mg l^−1^). The photoperiod was set to 12 hours light –12 hours dark. During the experiment both RAS and AS were operated as fully closed systems.

### Collection of gut and water samples

On each sampling day ten larvae per tank were collected for microbial community analysis. The larvae were euthanized with 0.6 g l^−1^ Tricaine Methanesulfonate (TMS, Crescent Research Chemicals, Phoenix, Arizona, USA), buffered with 0.12 g l^−1^ sodium bicarbonate in water originating from the corresponding rearing tank. Subsequently, larvae were rinsed with 70% ethanol and sterile water before dissecting out aseptically the gut under a dissection microscope. Whole gut samples were flash frozen in liquid nitrogen and stored individually at −80°C until subsequent analyses.

All tools and dissecting surfaces were disinfected with chloramine-T (Halamid-d, Veip BV, The Netherlands) and 70% ethanol between dissections. In addition, the tools were always held in a propane gas flame before use.

From each tank, 250 mL water samples were collected at the same time of gut sampling. The water was filtered through 0.45 µm (type HAWP) and 0.22 µm (type GTTP) membrane filters (Millipore - Isopore) using a vacuum apparatus.

The microbiota in the water and gut was analyzed using denaturing gradient gel electrophoresis (DGGE) of PCR-amplified 16S ribosomal RNA (rRNA) gene fragments. One water and 3 gut samples were taken from each of the 15 tanks and analyzed by using PCR-DGGE on days 0, 7, 14, 28 and 42. In addition, 1 water and 3 gut samples were taken from 2 replicate tanks of each RAS and from 2 AS tanks. Those samples were analyzed by 454 pyrosequencing of partial 16S rRNA genes on days 7 and 42. Samples that were analyzed with 454 pyrosequencing were a subset of the sample set that was analyzed with PCR-DGGE.

### Genomic DNA isolation

DNA was extracted from larval gut samples using the DNeasy Blood & Tissue Kit (Qiagen, Venlo, Netherlands) according to the manufacturer’s protocol with the following modifications: The gut samples were added to 180 µL enzymatic lysis buffer and incubated at 37°C for 1 hour. Furthermore, 40 µl proteinase K and 180 µL ATL buffer were added to improve cell lysis, and the mix was incubated for 1.5 h at 55°C. Cell-lysis was further optimized by performing an additional step in which gut tissue was homogenized in 200 ml AL buffer (Qiagen) with the aid of a custom bead mix (4 glass beads 2–3 mm, 0.5 g zirconia beads 0,1 mm) (MO-BIO Carlsbad, CA USA) and using the FastPrep instrument (QBioGene, Irvine, CA, USA) for 1 min at 6,000 rpm. The samples were eluted twice in 50 µl AE buffer. DNA concentration was measured with a NanoDrop ND-1000 spectrophotometer (NanoDrop Technologies, Wilmington, DE), and DNA samples were stored at −20°C until use.

For DNA extraction from water samples, the FastDNA SPIN kit for soil (MP Biomedicals, Ohio, USA) was used. The DNA was extracted from water membrane filters. Briefly, homogenization was achieved by addition of 978 µL sodium phosphate and 122 µl MT buffer, and the cell lysis in the lysing matrix was enhanced by a bead beating step of 40 s at 6000 rpm. DNA purification was achieved by addition of 1 mL silica binding matrix and 500 µL SEWS-M (salt ethanol wash) followed by centrifugation at 14,000 g for 5 min. The DNA was eluted by the addition of 50 µL DES (DNA elution solution ultra-pure water) and incubated at room temperature for 5 min. Subsequently, the DNA was collected by centrifugation at 14,000 g for 5 min. For more details see instructions given by the manufacturer.

### PCR-DGGE analysis

Target fragments of the bacterial 16S ribosomal RNA gene were amplified from the extracted DNA by PCR by using the following cycling conditions: 95°C for 2 min, followed by 35 cycles consisting of 95°C for 30 s, 53°C for 40 s and 72°C for 1 min and then a final 5 min extension step at 72°C. Samples were cooled to 4°C. PCR for DGGE was performed by using primers L1401-R (5′-CGGTGTGTACAAGACCC-3′) and U968-F (5′-CGCCCGGGGCGCGC CCCGG GCGGGGCGGGGGCACGGGGGGAACGCGAAGAACCTTAC-3′) fitted with a GC-clamp [16]. The PCR reaction mixture consisted of Phusion HF buffer, 0.2 µM of each primer, 200 µM of each dNTP, and 1 unit of Phusion Hot Start II High Fidelity Polymerase. To the 50 µl reactions 20–50 ng of DNA was added. Five µl of all PCR products were visualized by gel electrophoresis using 1% agarose gel with ethidium bromide to check the quality. DGGE analysis of PCR amplicons was performed as described previously [17] using the DCode system (Bio-Rad Laboratories, Hercules, CA). Polyacrylamide gels consisted of 8% (vol/vol) polyacrylamide (37.5∶1 acrylamide-bisacrylamide) in 0.5xTris-acetate-EDTA. A denaturing acrylamide containing 7 M urea and 40% formamide was defined as 100%. The gels were poured from the top by using a gradient maker (Econopump; Bio-Rad, La Jolla, CA) and pumping the solution at a speed of 4.5 ml min^−1^. A gradient from 30 to 60% was used for the separation of the PCR amplicons. Electrophoresis was performed for 16 h at 85 V in a 0.5xTris-acetate-EDTA buffer at a constant temperature of 60°C. Subsequently, gels were stained with AgNO_3_ according to the method described by Sanguinetti et al. [18].

### 454 Pyrosequencing

For more detailed 16S rRNA gene-based microbial composition profiling, barcoded amplicons from the V1–V2 region of 16S rRNA genes were generated by PCR using the 27F-DegS primer [19] that was appended with the titanium sequencing adaptor A and an 8 nucleotide sample-specific barcode [20] at the 5′ end. As a reverse primer, an equimolar mix of two primers 338R I and II [21] was used that carried the titanium adaptor B at the 5′ end. Extracted DNA was diluted to a concentration of 20 ng µl^−1^ based on Nanodrop readings. PCR was performed using a GS0001 thermocycler (Gene Technologies, Braintree, United Kingdom). The PCR mix (100 µl final volume) contained 20 µl of 5× HF buffer (Finnzymes, Vantaa, Finland), 2 µl 10 mM (each nucleotide) PCR-grade Nucleotide Mix (Roche Diagnostic GmbH, Mannheim, Germany), 1 µl of Phusion hot start II High-Fidelity DNA polymerase (2 U/µl) (Finnzymes), 500 nM of the reverse primer mix and the forward primer (Biolegio BV, Nijmegen, The Netherlands); 2 µl (i.e. 40 ng) template DNA and 65 µl nuclease free water. PCR was performed under the following conditions: 98°C for 30 s to activate the polymerase, followed by 30 cycles consisting of denaturation at 98°C for 10 s, annealing at 56°C for 20 s, and elongation at 72°C for 20 s, and a final extension at 72°C for 10 min. Twenty µl of the PCR products were analyzed by 1% (w/v) agarose gel electrophoresis in the presence of 1× SYBR Safe (Invitrogen, Carlsbad, CA, USA) and purified from gel using the High Pure PCR Cleanup Micro Kit (Roche Diagnostics) according to manufacturer’s instructions. DNA concentrations of gel-purified amplicons were measured by a Nanodrop ND-1000 spectrophotometer, and purified PCR products were mixed in equimolar amounts, run again on an agarose gel and subsequently excised and purified using a DNA gel extraction kit (Milipore, Billerica, MA, USA). Nucleotide sequences were generated by pyrosequencing using an FLX genome sequencer in combination with titanium chemistry (GATC-Biotech, Konstanz, Germany). Pyrosequencing data were deposited at the European Bioinformatics Institute in the sequence read archive under study accession number PRJEB4462 and sample accession numbers ERS343984– ERS344037.

The 454 pyrosequencing analysis was paired to the DGGE data by using samples collected from 2 replicate tanks on day 07 and 42, for the 2 RAS and 2 AS. We used both complementary methods for the characterization of spatiotemporal variation in the microbial communities in order to evaluate whether the outcome was consistent and comparable between methods, allowing for more general statements regarding the consequences for study design. Although pyrosequencing provided also useful direct sequence information with respect to the composition and ecology of the microbial communities in the samples, this was beyond the scope of this study and will be addressed in a separate paper.

### Normalization between DGGE gels

On every DGGE gel a standard reference marker consisting of an amplicon mix of 10 different cloned bacterial 16S rRNA genes was included at 3 different positions, for digital gel normalization. These 10 fragments of the reference marker produced a known distinctive pattern defined by the position of the bands. The designation of the inter-gel band classes was based upon their relative position on the profile compared with the standard reference used, as described above. An overall comparison of the reference markers between all gels showed that all markers from the 15 gels clustered together, with a similarity higher than 95% and regardless of the gels that they belonged to, indicating that a valid comparison in community fingerprints was possible also between multiple gels. Using a standard reference marker to allow intra and inter-gel comparisons, has been suggested elsewhere [22]–[32]. In addition to that, the inter-gel variation among profiles was tested at the beginning of the DGGE analysis. To this end, randomly selected samples from 2 or 3 different gels were selected and re-loaded into a single gel. DGGE gels (Figures S2, S3 and S4) showed that samples were clearly grouped based upon their band pattern and not upon the gel they belonged to, allowing for a fair inter-gel comparison.

### Data handling and statistical analysis

DGGE patterns were analysed with Bionumerics software 5.1 (Applied Maths, St-Martens-Latem, Belgium) following the manufacturer’s instructions. The patterns were normalized and individual bands were initially marked automatically (5% minimum profiling), followed by visual inspection and manual correction whenever necessary. For automatic band matching the position tolerance of the fingerprints was set to 1% (percentage of the pattern length) maximum shift between two bands. Optimization for the best possible matching was set to a maximum allowable shift of 0.5%. The band-classes were arbitrarily generated in a global alignment of all entries (DGGE lanes) of combined DGGE gels, by tracing common bands across different profiles. The designation of the band-classes was based on their position in the profile compared with the reference marker used as a normalization standard, to ensure gel-to-gel comparability. The bands were furthermore inspected manually for consistency. As measure of relative abundance, relative intensity of each band within individual DGGE profiles was used. Subsequently, data were square root transformed to decrease the importance of the most dominant bands in the subsequent analysis [33].

Pyrosequencing data were analyzed using the QIIME 1.5.0 pipeline [34], and quality filtering (de-noising) was performed as follows. Low quality sequences were removed using default parameters (i. reads with fewer than 200 or more than 1000 nucleotides; ii. reads with more than 6 ambiguous nucleotides, homopolymer runs exceeding 6 bases, reads with missing quality scores and reads with a mean quality score lower than 25; iii. reads with mismatches in the primer sequence), and operational taxonomic units (OTUs) were identified at the 97% identity level. Representative sequences from the OTUs were aligned using PyNAST [35]. The taxonomic affiliation of each OTU was determined using the RDP Classifier at a confidence threshold of 80% against the 12_10 Greengenes core set [36]. Possible chimeric OTUs were identified using QIIME’s ChimeraSlayer and removed from the initially generated OTU list, producing a final set of non-chimeric OTUs.

For the DGGE data, there were five possible factors in the experimental design: “origin” (two levels; gut and water; fixed), “date” (five levels; day 0, 7, 14, 28, 42; fixed), “system type” (two levels; RAS and AS; fixed), “replicate system” (2 RAS or 5 AS; nested in system type: random) and “tank” (five levels, tank 1, 2, 3, 4 and 5, nested in replicate system: random). Because of the highly skewed distribution of bacterial species and the large number of zeros contributed by rare species, the assumption of multivariate normal distribution was unrealistic. For that reason a permutation-based multivariate ANOVA (PERMANOVA) was used to analyze the data set [37]. This method allows multivariate data to be analyzed on the basis of any distance or dissimilarity measure. The distance matrix was based on Bray Curtis dissimilarity [38] due to its desirable properties when compared to other distance measures for analyzing environmental data. For example, the Bray Curtis coefficient does not increase the similarity between two samples when a common species absence occurs [39] which is a very useful property when analyzing biological assemblage data with many zeros. For each term in the analysis, 9999 permutations of raw data units were performed to calculate P values, and when there were not enough possible permutations a Monte Carlo sample was drawn from the theoretical asymptotic permutation distribution [40].

In addition to PERMANOVA, analysis of similarities (ANOSIM) was used to give an insight into the degree of separation between the tested groups of samples. ANOSIM tests the null hypothesis that the average rank similarity between samples within a group is the same as the average rank similarity between samples belonging to different groups. The analysis produces an R statistic that generally ranges from 0 to 1 [41]. An R of 1 indicates complete separation whereas an R of 0 indicates that the null hypothesis is true. The statistical significance of R statistic is assessed by random permutations of the group membership to obtain the empirical distribution of R under the null-model and is free of any assumption of normality [39].

Although neither PERMANOVA nor ANOSIM explicitly assume common variances among groups, they are both sensitive to differences in multivariate dispersion. To test the hypothesis of equal within group dispersion (for both methods) PERMDISP analysis as a multivariate non-Euclidean equivalent to traditional Levene’s test was used [42]. The analysis was used for two reasons: i. as a complementary test to avoid any kind of misinterpretation of the outcome of the two previous methods mostly due to type II error, and ii. to give insight of within and between groups variation. Homogeneity of dispersion among groups was calculated as an average distance (±SE) of group members (samples) from the group’s centroid. PERMDISP was used to test the null hypothesis of no difference between groups dispersion. Significant effects on group dispersion were tested for “tank”, “replicate system”, ”system type” and “date”.

Non metric multi-dimensional scaling (nMDS) was performed to represent the samples in a low dimensional space in a way that relative distances of all points are in the same rank order as the relative dissimilarities of the samples as measured by Bray Curtis index. “Stress” values in nMDS indicate how well the multidimensional relationships among the samples are represented in the low dimensional space.

Hierarchical agglomerative clustering with group average linking (based on Bray Curtis similarity) was used to identify “natural groupings” (meant as non-predefined groups) of samples, in such a way that partitioning of groups indicates differences in the microbial community between them. To verify cluster patterns even for the most clearly congregated samples, cluster analysis was used in combination with nMDS plots, as well as the results from the estimation of the components of variation in PERMANOVA.

All statistical analyses were performed by using the multivariate statistical software package Primer V6 (Primer-E Ltd, Plymouth, UK).

## Results

During the experiment, the water quality was maintained within preset limits resulting in above 99% survival. Fish grew on average 11.17±0.06% g in RAS and 11.03±0.05% body weight d^−1^ in AS with a feed conversion ratio of 0.64±0.01 in RAS and 0.70±0.01 (± SD) in AS. The final weight reached was 1.24±0.03 g in RAS and 1.17±0.03 g in AS. No significant differences were observed between replicate systems, neither for water quality nor for fish growth (P>0.05).

### Overall contribution of factors in microbial dynamics

**Figure 1:**
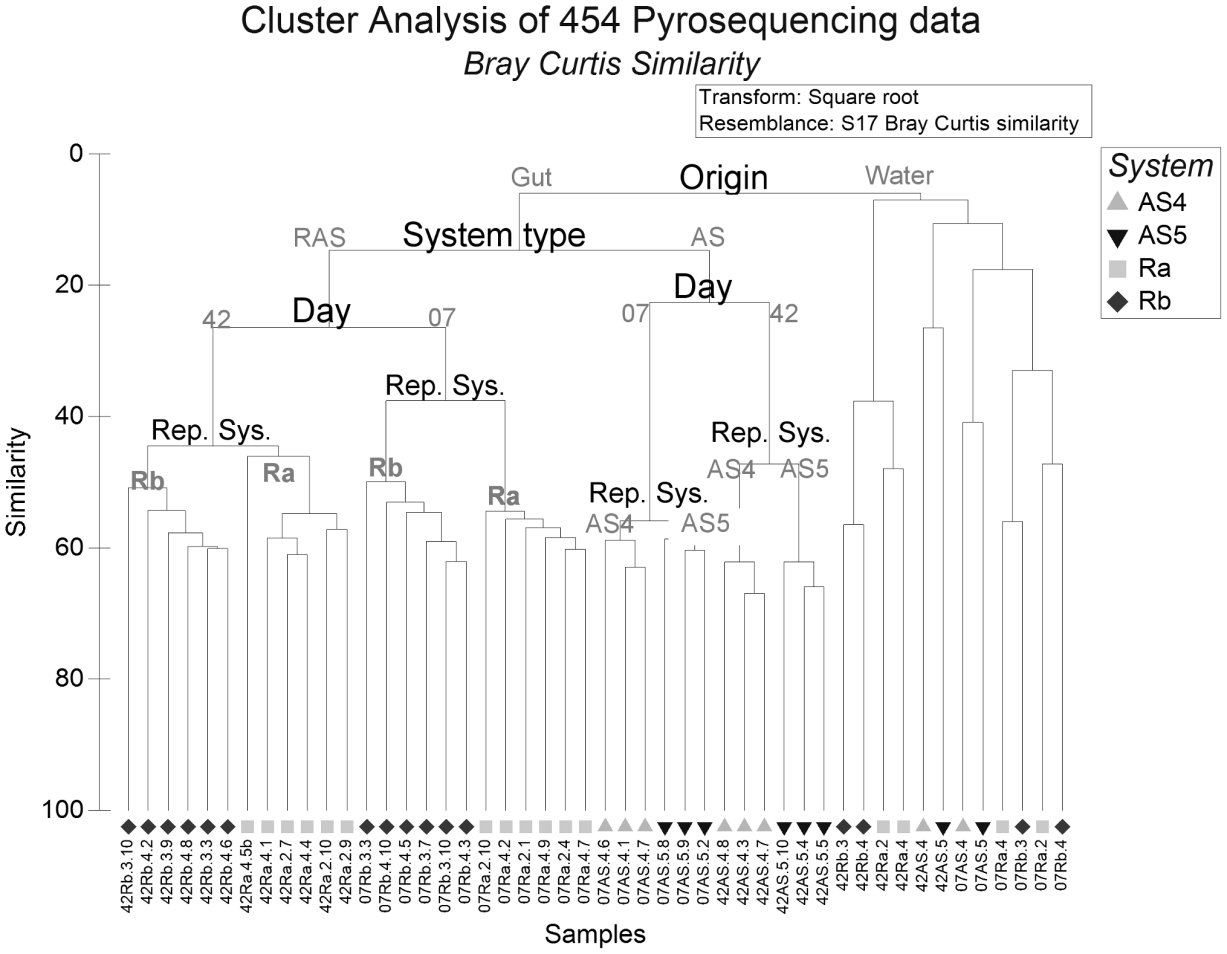
Hierarchical clustering with Unweighted Pair Group with Arithmetic Mean (UPGMA) linkage of gut and water samples based on 454 data. On y-axis: similarity percentage based on Bray Curtis similarity, on x-axis: all individual samples of gut (left) and water (right). D07 and 42: sampling day 7 & 42, AS 4 & 5: replicate active suspension systems 4 & 5, Ra and Rb: replicate recirculating system a and b. Numbers 2, 4 & 3, 4: replicate tanks from Ra and Rb respectively. Last digits following the tank number indicate the number of replicate fish in each tank. Since only one water sample was taken from each tank, the last digits were omitted from water sample’s ID. (e.g. 42Rb.3.10: Gut of day 42, from recirculating system b, tank 3, fish 10 whereas 07Ra.2: Water of day 7, from recirculating system a, tank 2).

PERMANOVA of DGGE data revealed significant effects of all main factors (“system type”, “replicate system”, “date” and “origin”) except for “tank” (Table 1). A similar picture emerged for the pyrosequencing data. The highest fraction of total variation was explained by the main factor “origin”, followed by “system type”, “date” and “replicate system” (Figure 1). Lowest similarity was observed between gut and water microbiota. Gut samples were separated into RAS and AS systems, confirming that system is the principal factor differentiating gut microbiota. In both systems, gut samples differed significantly between day 07 and 42. Differences between gut samples from “replicate systems” were less pronounced than for “origin”, “system type” and “date”.

**Table 1:** Overall PERMANOVA test based on DGGE data for main experimental factors.

Factor	df	Pseudo-F	P(MC)
**System type**	1	5.9632	0.0001
**Date**	4	4.9623	0.0001
**Origin**	1	9.1458	0.0001
**Replicate system**	5	5.6948	0.0001
**Tank**	8	1.0661	0.2891

P values are based on 9999 Monte Carlo (MC) permutations. Effects of the interaction terms are not shown in the table.

### Variation in gut and water microbiota from replicate systems

#### Recirculation systems (RAS)

**Figure 2:**
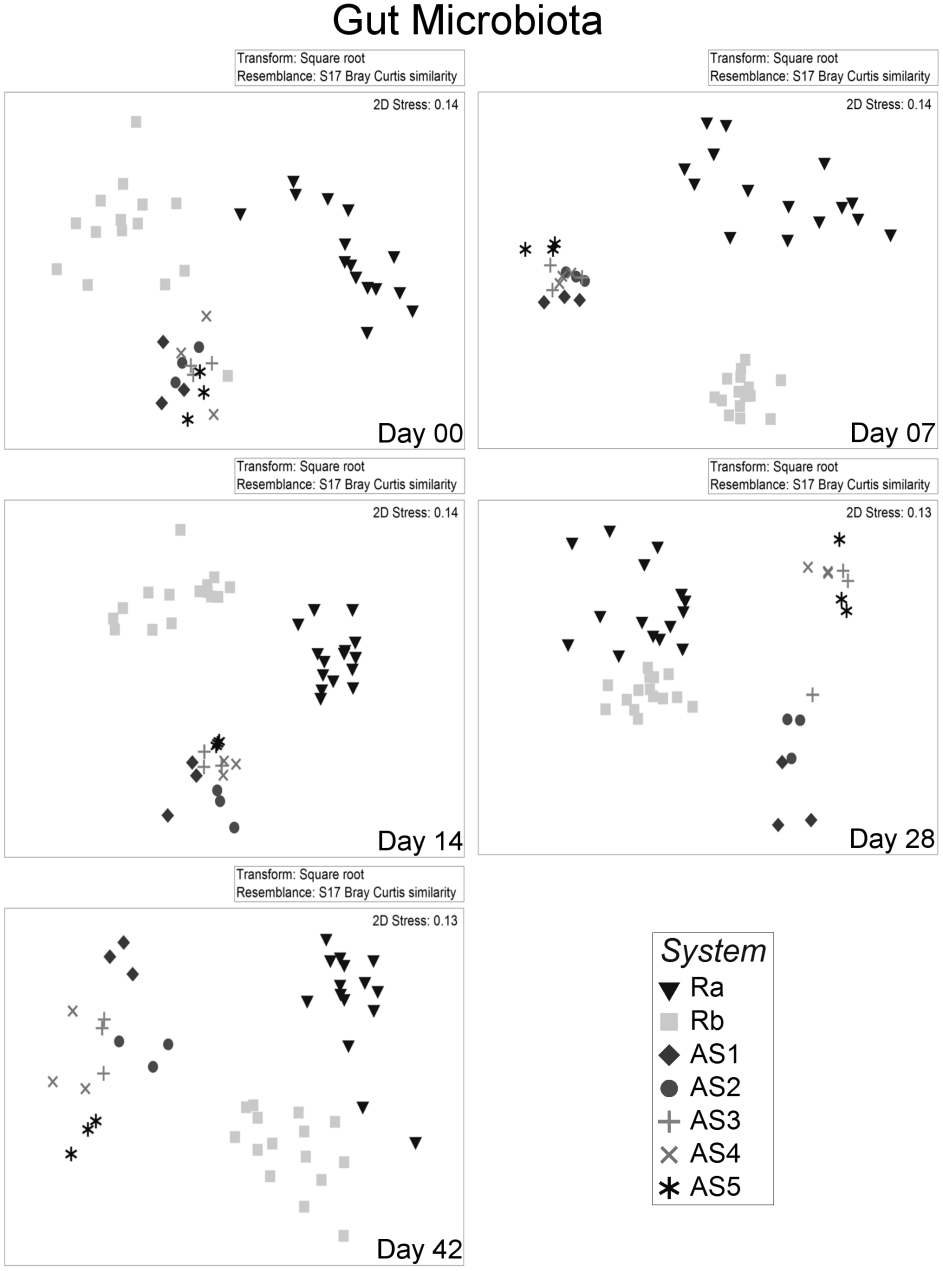
Non metric dimensional scaling (nMDS) of gut microbiota from individuals reared in different systems over time. Each point represents the gut microbiota of one individual. Plots are based on Bray Curtis distance after square root transformation of relative abundance DGGE data. D00, 07, 14, 28, 42: sampling days 0, 7, 14, 28 & 42 respectively, AS1-5: replicate active suspension system 1 to 5, Ra & Rb: replicate recirculating system a & b. Stress values are reported for the two dimension and are indicative of the goodness of fit of data into the plot.

**Figure 3:**
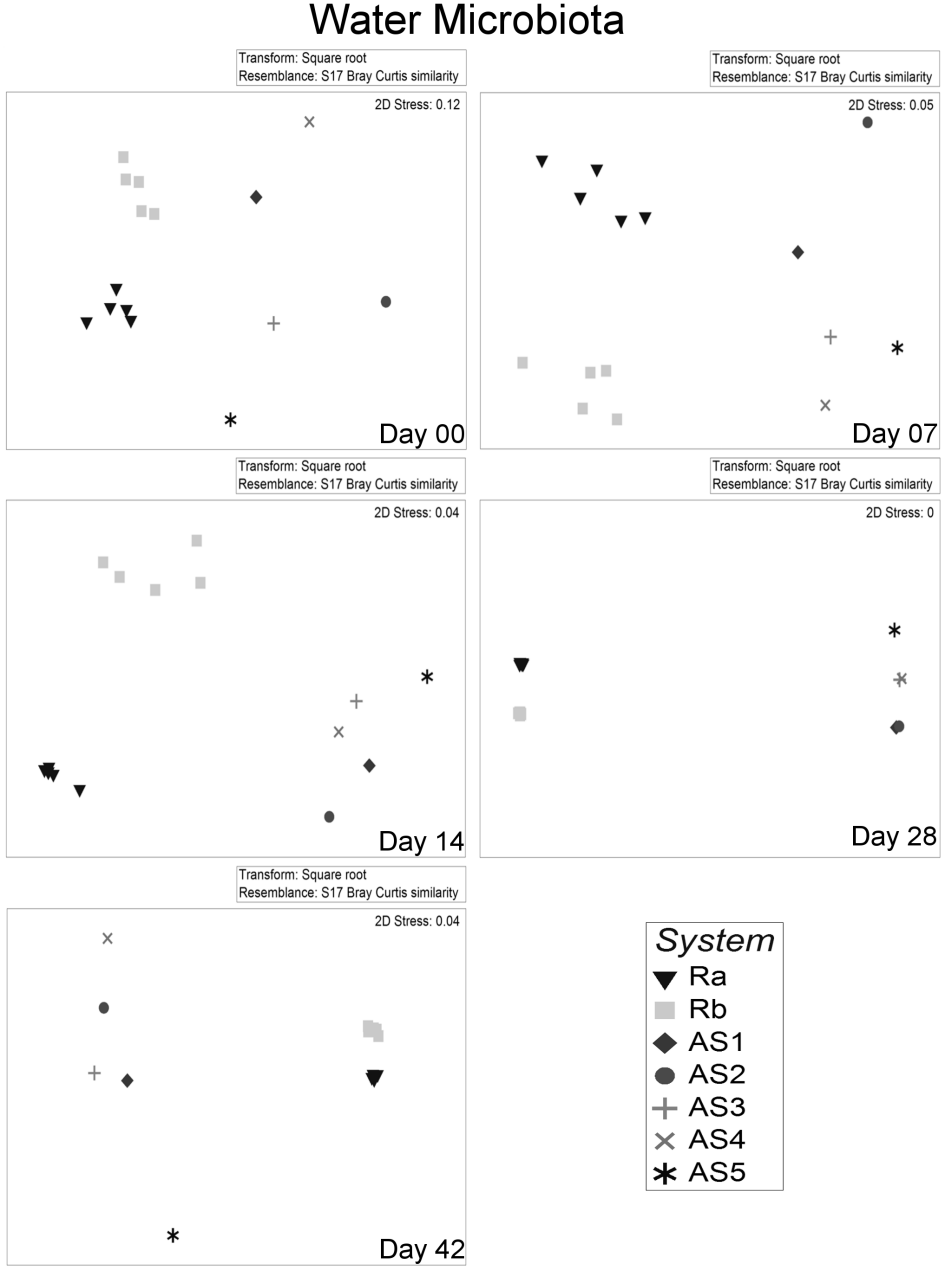
Non metric dimensional scaling (nMDS) of water microbiota from different systems over time. Each point represents the water microbiota from each tank. Plots are based on Bray Curtis distance after square root transformation of relative abundance DGGE data. D00, 07, 14, 28, 42: sampling days 0, 7, 14, 28 & 42 respectively, AS1-5: replicate active suspension system 1 to 5, Ra & Rb: replicate recirculating system a & b. Stress values are reported for the two dimensions and are indicative of the goodness of fit of data into the plot.

Gut microbiota of Ra and Rb differed consistently (P<0.05; Table 2) during the 42 days experimental period, irrespective of the choice of analytical method (DGGE and 454) or statistical test (PERMANOVA or ANOSIM). nMDS ordination plots of the DGGE data showed a clear distinction between the microbiota of individuals reared in Ra and Rb for all dates (Figure. 2), confirming the R statistic in ANOSIM (Table 2). The comparison of water microbiota between Ra and Rb also differed consistently (Figure 3).

**Table 2:** Pairwise comparisons between replicate systems for RAS and AS separately, based on DGGE and 454 pyrosequencing data sets.

Statistical test	Analytical Method		D00		D07		D14		D28		D42	
			AS1-5	Ra-Rb	AS1-5	Ra-Rb	AS1-5	Ra-Rb	AS1-5	Ra-Rb	AS1-5	Ra-Rb
**Perm. ANOVA**	**GUT**	**DGGE**	2/10	P:0.0001	6/10	P:0.0003	6/10	P:0.0001	7/10	P:0.0003	7/10	P:0.0001
		**454**			P:0.2079	P:0.0025					P:0.0242	P:0.016
**ANOSIM**		**DGGE**	R:0.428	R:0.925	R:0.787	R:0.772	R:0.542	R:0.922	R:0.745	R:0.596	R:0.907	R:0.862
			P:0.0005	P:0.0001	P:0.0001	P:0.0001	P:0.0001	P:0.0001	P:0.0001	P:0.0001	P:0.0001	P:0.0001
		**454**			R:1	R:1					R:1	R:0.846
					P:0.1	P:0.002					P:0.1	P:0.002
**Perm. ANOVA**	**WATER**	**DGGE**		P:0.0002		P:0.0212		P:0.0002		P:0.0001		P:0.0008
**ANOSIM**				R:0.996		R:0.972		R:1		R:1		R:0.988
				P:0.008		P:0.008		P:0.008		P:0.008		P:0.008

Tests were performed by sample type per sampling day, with P values for each comparison from two different statistical tests (PERMANOVA and ANOSIM). ANOSIM results are complementary to the PERMANOVA to provide information on the degree of separation between groups, suggested by R statistic. The 2/10, 6/10 & 7/10 indicate the number of significant out of the total available comparisons. D00, 07, 14, 28, 42: sampling day 0,7,14,28 & 42 respectively, AS1-5: replicate active suspension system 1 till 5, Ra & Rb: replicate recirculating system a & b. P values are based on 9999 Monte Carlo permutations.

#### Active suspension systems (AS)

Gut microbiota was different between individuals reared in different AS systems at day 07 and day 14, whereas this was not the case on day 0 (Table 2). On day 07, the five AS systems were not statistically different when using PERMANOVA. Nevertheless, out of 10 possible pairwise comparisons, six comparisons indicated significant differences between the five AS systems (P values of each of the pairwise tests are not shown). ANOSIM’s R statistic suggested a clear distinction between AS systems for both DGGE and 454 data on day 07. At day 14, the same pattern emerged (6/10 pairwise tests showed differences, and ANOSIM’s R statistic was 0.542). At days 28 and 42, AS systems were different (Table 2). Due to lack of replicate samples pairwise comparisons between water samples of AS systems were not possible.

To evaluate differences between RAS and AS systems, pooled data of Ra and Rb were compared with pooled data from AS. Pairwise comparisons (Table 3) for PERMANOVA and ANOSIM showed that microbiota in gut or water were different between RAS and AS from day 07 onwards (P<0.001).

**Table 3:** Pairwise comparisons between RAS and AS systems based on DGGE and 454 pyrosequencing data sets.

Statistical test	Analytical Method		RAS vs. AS				
			D00	D07	D14	D28	D42
**Perm. ANOVA**	GUT	DGGE	P:0.0710	P:0.0022	P:0.0202	P:0.0063	P:0.0099
		454	NA	P:0.0152	NA	NA	P:0.0056
**ANOSIM**		DGGE	R:0.244 P:0.0020	R:0.731 P:0.0001	R:0.496 P:0.0001	R:0.881 P:0.0001	R:0.872 P:0.0001
		454	NA	R:1 P:0.0001	NA	NA	R:1 P:0.0001
**Perm. ANOVA**	WATER	DGGE	P:0.0983	P:0.008	P:0.0187	P:0.0136	P:0.0062
**ANOSIM**			R:0.820 P:0.0010	R:0.944 P:0.0010	R:0.940 P:0.0003	R:1 P:0.0020	R:0.990 P:0.0020

Tests were performed by sample type per sampling day, with P values for each comparison from two different statistical tests (PERMANOVA and ANOSIM). ANOSIM analysis is complementary to PERMANOVA as it provides information on the degree of separation between groups, suggested by R statistic. N.A.: No pyrosequencing data available for that day. D00, 07, 14, 28, 42: sampling day 0,7,14,28 & 42 respectively, AS1-5: replicate active suspension system 1 till 5, Ra & Rb: replicate recirculating system a & b. P values are based on 9999 Monte Carlo permutations.

### Variation in gut microbiota of larvae reared in different tanks

The extent of variation in gut microbiota of animals reared in replicate tanks of the same recirculation system was evaluated based on pairwise comparisons of profiles obtained by either DGGE (five tanks per system) or 454 pyrosequencing (two tanks per system). On day 0, gut microbiota was similar (P>0.05 for all pairwise comparisons) between all replicate tanks in either Ra or Rb. For all subsequent sampling days, the majority (72% of all possible comparisons) of the pairwise tests indicated that gut microbiota was not different between replicate tanks (P>0.05, Table 4).

**Table 4:** Pairwise comparisons between individuals reared in replicate tanks of the same system.

Statistical test	Analytical Method	Ra tanks	D00	D07	D14	D28	D42	Rb tanks	D00	D07	D14	D28	D42
**PERMANOVA**	**DGGE**	***6–7***	0.286	0.429	0.124	0.130	**0.029**	***11–12***	0.709	0.099	0.273	**0.047**	0.520
		***6–8***	0.535	0.494	0.155	**0.026**	**0.009**	***11–13***	0.631	0.168	0.383	0.533	0.737
		***6–9***	0.217	**0.042**	**0.026**	0.081	**0.011**	***11–14***	0.330	0.052	0.068	**0.044**	0.493
		***6–10***	0.365	**0.017**	**0.043**	0.298	**0.009**	***11–15***	0.269	**0.014**	0.091	0.122	**0.024**
		***7–8***	0.338	0.260	0.295	0.177	0.298	***12–13***	0.272	0.266	0.325	0.547	0.768
		***7–9***	0.150	**0.015**	**0.027**	0.302	0.294	***12–14***	0.212	0.071	**0.013**	**0.049**	0.341
		***7–10***	0.126	**0.008**	0.083	0.233	0.105	***12–15***	0.114	**0.017**	**0.028**	0.228	**0.024**
		***8–9***	0.453	0.055	**0.037**	0.081	0.243	***13–14***	0.604	0.149	0.151	0.557	0.596
		***8–10***	0.458	**0.025**	0.092	0.068	0.147	***13–15***	0.383	0.055	0.187	0.653	0.074
		***9–10***	0.542	0.067	0.665	0.432	0.230	***14–15***	0.319	0.144	0.408	0.104	0.079
	**454**	***7–9***	NA	0.432	NA	NA	0.411	***13–14***	NA	0.452	NA	NA	0.432

Analysis was performed both on DGGE and 454 pyrosequencing data sets. D00, 07, 14, 28, 42: sampling day 0,7,14,28 & 42 respectively, Ra & Rb: replicate recirculating system a & b. Tank numbers 6–10 & 11–15, refer to replicate tanks of Ra and Rb, respectively. P values are based on 9999 Monte Carlo permutations. P values<0.05 are highlighted in bold.

### Temporal dynamics in gut and water microbiota

The temporal dynamics in gut microbiota was tested separately for Ra and Rb and for AS (AS1 to AS5) systems. PERMANOVA on DGGE data revealed a significant impact of time on gut microbiota (Table 5). Pairwise comparisons of DGGE patterns obtained for consecutive sampling dates were performed, and for all systems there was a significant time effect for every pairwise comparison (Table 5, P<0.05). Also for water samples, the temporal variation of microbiota was tested separately for Ra and Rb. At “system type” level, pairwise comparisons of samples taken at consecutive dates indicated that the water-associated microbiota was different between dates (P<0.05) (Table 5).

**Table 5:** Pairwise comparisons between consecutive days for RAS and AS replicate systems separately.

Statistical test		Groups (dates)	AS					RAS	
			AS1	AS2	AS3	AS4	AS5	Ra	Rb
			P (MC)	P (MC)	P (MC)	P (MC)	P (MC)	(P)	(P)
**PERMANOVA**	**GUT**	**00, 07**	0.010	0.001	0.005	0.010	0.003	0.002	0.006
		**07, 14**	0.009	0.013	0.002	0.001	0.001	0.001	0.010
		**14, 28**	0.008	0.006	0.015	0.001	0.003	0.002	0.002
		**28, 42**	0.010	0.005	0.033	0.008	0.005	0.005	0.004
**ANOSIM**		**Global R (all groups)**	R:1	R:0.997	R:0.833	R:1	R:1	R:0.906 P:0.001	R:0.938 P:0.001
**PERMANOVA**	**WATER**	**Groups (dates)**	**AS**					**Ra**	**Rb**
		**00, 07**	0.0079					0.0003	0.0017
		**07, 14**	0.0041					0.0006	0.0009
		**14, 28**	0.0028					0.0001	0.0001
		**28, 42**	0.0091					0.0003	0.0004
**ANOSIM**		**Global R (all groups)**	R:0.788/P: 0.0001					R:0.999 P:0.0001	R:0.989 P:0.0001

Analysis is based on DGGE data sets. Tests were performed by sample type, with P values for each comparison from two different statistical tests (PERMANOVA and ANOSIM). ANOSIM results are complementary to the PERMANOVA as they provide information on the degree of separation between groups, suggested by R statistic. D00, 07, 14, 28, 42: sampling day 0, 7, 14, 28 & 42 respectively, AS1-5: replicate active suspension system 1 till 5, Ra & Rb: replicate recirculating system a & b. P values are based on 9999 Monte Carlo permutations.

### Within group dispersion as a measure of dissimilarity between individuals

**Figure 4:**
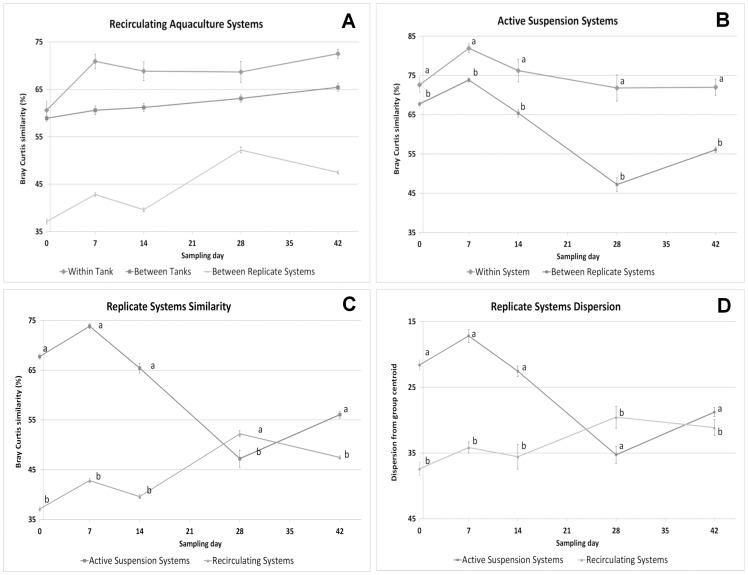
Bray Curtis similarity (%) (A, B & C) and dispersion from centroid based on Euclidian distance (D) for each sampling day. Points represent mean values of gut microbiota between individuals reared either in the same tank (Within tank), replicate tanks (Between tank) or between systems (Between System). (A): Comparison for Recirculating Aquaculture Systems (RAS). (B) Comparison for Active Suspension (AS). (C) Comparison between RAS and AS Systems & (D) Dispersion of samples from group centroid in RAS and AS systems. Error bars show standard error. Different data labels (a, b and c) per sampling day indicated significant difference (P<0.05) based on one-way ANOVA and Bonferroni ranking test for A, B & C and permutation dispersion test for D, (P(perm)<0.05).

One way ANOVA on Bray Curtis similarity indicated a clear tank or system effect, with individuals being more similar within than between tanks. Between replicate systems the similarity was even lower (Figure 4 A & B). When comparing similarity between individuals of replicate systems, AS replicate systems were more similar than RAS systems (Figure 4 C). These differences in Bray Curtis similarity concurred with differences in group dispersion (Figure 4 D). Mean group dispersion indicated that microbiota of individuals reared in AS systems were less dispersed, thus more similar, than for individuals reared in RAS until culture day 28 (Figure 4 C & D). By day 42 the differences in dispersion became non-significant (P(perm) = 0.095). There was a significant overall time effect on the group dispersion within system. In AS systems dispersion increased (individuals became less similar) over time (AS: F = 26.205 (df_system_: 4, df_time*samples_: 70), P(perm) = 0.001), whereas in RAS systems dispersion decreased (RAS: F = 11.683 (df_system_: 4 df_time*samples_: 145), P(perm) = 0.0001), until the dispersion within the two systems converged on day 28 and 42 (Figure 4 D). These trends were confirmed by the Bray Curtis similarity means over time for AS and RAS (Figure 4 C).

## Discussion

In this study we characterized the spatio-temporal variation in the gut microbiota of tilapia larvae, reared in two different types of aquaculture systems. As was mentioned in the introduction, a subset of samples was analyzed using two different analytical methods, namely DGGE and pyrosequencing of PCR-amplified 16S rRNA gene fragments. The comparison revealed that data obtained by both methods were not contradicting each other. DGGE as a method has some specific limitations, for instance, the separation of relatively small DNA fragments, the co-migration of DNA fragments with different sequences, the detection of hetero-duplex molecules and the limited sensitivity of detection of rare community members [43]. In addition, multiple comparisons among different DGGE gels might lead to a false positive conclusion due to high gel to gel variation [17], [44]. However, in this study these errors were small and did not jeopardize the broader picture, as it was also confirmed by the pyrosequencing data analysis.

### Differences in gut and water microbiota in different tanks

The results showed that variation in individual gut microbiota within tanks was similar to the variation between tanks. This can probably be explained by the fact that larvae shared the water source and feed. To this end, it is interesting to note that pairwise comparisons indicated that the microbiota in larvae reared in replicate tanks in the same RAS were mostly similar, but not in all cases (Table 4). Bakke et al. [45] pointed out that when sampling a few fish in only two replicate tanks, on one occasion gut microbiota differed between two tanks, whereas on another occasion gut microbiota was similar between two other tanks. When sampling only a few individuals in only two tanks the power of the analysis is low. We sampled only three individuals per tank resulting in 10 possible unique permutations. This allows only for a maximum significance level of 10% and in such cases Monte Carlo permutation was used. Nevertheless, unique permutation based P values are preferred when the minimum significance level drops below 1%; this will be realized when sampling a minimum of individuals per tank. Anderson et al. [46] suggested that examining average within/between group dissimilarities and dispersion, as well as using unconstrained ordination plots, helps to reveal the nature of differences among groups detected by PERMANOVA. In our case, nMDS plots of the DGGE data and the cluster analysis of the pyrosequencing data did not show a clear separation of gut microbiota between larvae reared in replicate tanks. Moreover, ANOSIM’s R statistic of tank pairwise comparisons was very low or even negative, also suggesting there is no tank effect on gut microbial communities. Bakke et al. [45], in contrast to our findings, reported differences in gut microbiota between replicate tanks. This might be due to cumulative differences in water microbiota between replicate tanks and variation in microbiota of daily fed live feeds as opposed to the pelleted commercial diet used in our study. Another reason might be that Bakke et al. [45] extracted DNA from whole cod larvae after homogenization. Although larvae were disinfected externally, the possibility of contamination cannot be excluded. In our study, fish guts from comparatively much larger tilapia larvae were dissected aseptically after sterilizing body surfaces, with lower risk for contamination. High within tank variation in gut bacteria of cod larvae was also reported by Fjellheim et al. [13]. Here too, larvae were fed live feeds, and whole larvae were used for DNA extraction. In addition, larvae were sampled only from one tank per treatment, and conclusions were drawn based on a combination of culture dependent and independent techniques. These results should be considered with caution, because the cultivability of microbiota varies with species composition.

### Differences in gut and water microbiota between replicate systems

Gut microbiota between replicate AS systems became different within one week (P<0.05), whereas gut microbiota of the individuals reared in Ra and Rb was different already from day 0 (P<0.05; 43.8% ±0.26 SE Bray Curtis similarity). Microbiota in water was also different (P<0.05) between Ra and Rb. Verschuere et al. [47] monitored the water microbial communities in three identical *Artemia* culture series, showing distinct microbial communities developing in each of them, suggesting differentiation is stochastic. This concurs with the observed differences of microbiota in gut and water between replicated RAS or AS systems in this study (Figure 1). On each sampling day, based on their gut microbiota, larvae reared in Ra differed from those reared in Rb. Similarly, larvae reared in AS4 and AS5 differed (Figure 1; P<0.05). This difficulty to replicate systems when studying individual gut microbiota makes experimental design challenging.

In our study, water quality parameters and fish growth were not significantly different between replicate systems (data not shown), yet their microbial communities differed. The observed differences in microbial composition do not necessarily imply differences in functionality [48]. Functional redundancy suggests that functional diversity of an ecosystem is additive when species are complementary, or decreases, when species share functions [49]. Our results suggest that different treatments (for example, testing dietary effects on gut microbiota) should preferably be tested in tanks within the same system, to reduce variation due to system replication.

### Differences in gut and water microbiota between different types of rearing systems

Except for day 0, water and gut microbiota differed between RAS and AS, suggesting a clear system effect. Larval growth, feed conversion and survival between RAS and AS were similar (data not shown), and it is thus safe to assume that observed differences in gut microbiota were not caused by growth related factors or health status of the larvae. Regarding water, rearing system type also affected microbial communities. Possible underlying mechanisms will be explored in a separate paper focusing on differences in bacterial community species composition based on pyrosequencing data.

One question is whether differences in gut microbiota can be explained by differences in water microbiota. Water microbiota, together with feed microbiota, have a large impact on gut microbiota in early life stages [3]. Bakke et al. [45] suggested that relatively small differences in water microbiota may impose significant differences in larval microbiota, and this might also be the case in our study. Cluster analysis of Bray Curtis similarity of relative abundance data showed that only 10% of the gut and water microbiota was overlapping (Figure 1). Nevertheless, species sub-dominant or even below the detection threshold in the water might be dominant in the gut, or vice versa.

The lack of significant differences in gut microbiota between RAS and AS on day 0 might be due to two reasons; i. high similarity between the microbial communities of the two systems or ii. high within system variation (dispersion). Anderson [46] suggested that PERMANOVA test should be used combined with a test of homogeneity of multivariate dispersion (PERMDISP). Our results showed that dispersion in RAS was significantly higher at day 0, (compared to the rest of the days), and this was most likely the reason that gut microbiota from larvae reared in RAS did not differ significantly from the ones reared in AS. This might as well explain why water microbial communities between the two systems did not differ on day 0, as PERMDISP confirms that water microbiota among the five different AS systems was more dispersed on day 0 than on any other day of the experiment.

### Temporal variation in gut and water microbiota

It is interesting to observe, that in spite of the enormous changes during early development, the effect of “date” was not the most pronounced among factors. The “date” effect to a large extend is linked to structural and functional changes of the gut during early development, including changes in the gut microbiota [50]. Changes could be induced by fluctuations in pH, gastric secretions and digestive enzymes activity, presence of bile salts, nutrients availability (from endogenous to exogenous feeding), as well as some stochastic events [3], [47].

**Figure 5:**
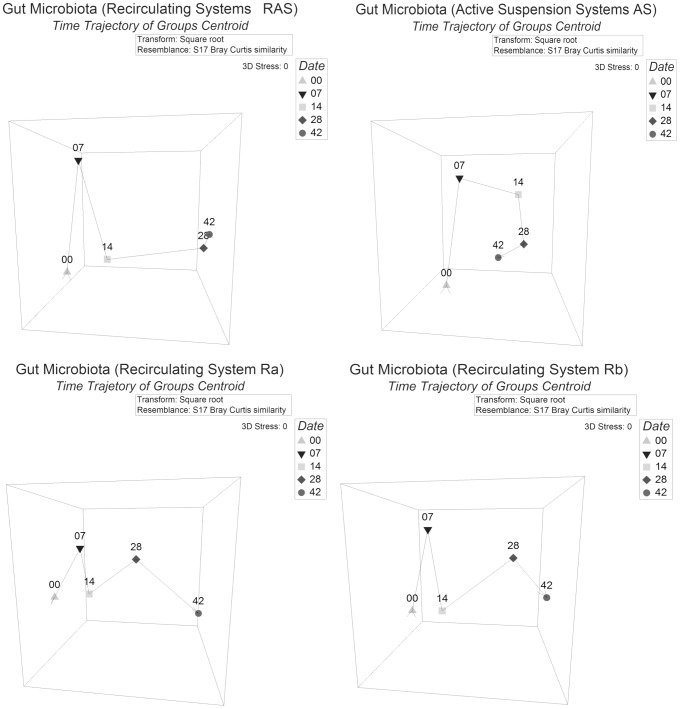
Three dimensional nMDS plots of gut microbiota from different systems over time (trajectory). Plots are based on Bray Curtis distance after square root transformation of relative abundance DGGE data. D00, 07, 14, 28, 42: sampling days 0, 7, 14, 28 & 42 respectively. Each point in the plots represents the group centroid and the shift of group average microbiota in time. Zero stress values for each plot are indicative of the fit due to the representation of the centroids. AS and RAS: active suspension and recirculating system, Ra & Rb: replicate recirculating system a & b.

When plotting the temporal trajectories of gut microbiota of larvae reared in RAS and AS, both systems clearly differed from day 07 onwards. While the trajectories of the five AS systems were very different, changing almost stochastically (plots not shown), the two replicate RAS trajectories were similar even if the two replicate RAS did not share the same water source (Figure 5). There was a clear distinction of day 0, 7 & 14 from days 28 and 42 for the RAS systems, whereas for the AS systems such a separation was not evident. This agrees with the observed overall (all five points) lower dispersion of AS gut samples compared to dispersion of RAS gut samples. This might be due to two reasons; i. Gut microbiota changed less over time in AS than in RAS; ii. Microbiota of individuals was more similar on each sampling day in AS than in RAS. A possible explanation is that in AS systems solids remain in the fish tank and most of the organic carbon and nitrogen is available for heterotrophic bacteria. These bacteria are abundant in high concentrations in the water reaching densities of 10^7^ CFU ml^−1^ [51]. Bacteria, protozoa, algae and zooplankton form bioflocs, which are directly available to fish [52]. Grazing on bioflocs might have caused gut microbiota in AS to be more uniform than in RAS.

## Conclusions

Microbiota in water and in larval guts between replicate systems was very different. When individuals share the same water, the rearing tank had a minor effect on gut microbiota. Compositional replication of the microbial communities at system level was not successful. Apparently, our understanding and control of underlying deterministic and stochastic factors is insufficient. This poses many challenges when researching treatment effects on gut or water microbiota. We recommend to investigate treatment effects on gut microbiota within the same system (fish share the same water source), rather than between replicate systems, unless systems can be replicated within treatment. Our results showed that gut microbiota of individuals between tanks of the same system did not differ, whereas between replicate systems they did. The observed rapid and stochastic changes of microbiota in gut and water over time, suggests that long term studies should be interpreted carefully. Observations of start and endpoint do not provide information about the temporal variation in between.

## Supporting Information

Figure S1 **Schematic overview of experimental factors and levels.** Five active suspension (AS) and 2 recirculating aquaculture systems (RAS) were used. The replicate RAS are named Ra and Rb; the replicate AS systems are named AS1 through AS5. Each RAS contained five tanks which shared the same water source. AS systems did not have sub-divisions. For DGGE analysis, three guts and water were sampled from each tank in RAS (10 tanks total) and each AS (5 systems) on sampling day 00, 07, 14, 28 & 42. Sub-sets of samples for DGGE of Ra2, Ra4, Rb3 and Rb4 (dark shaded tanks), and active suspension systems AS4 and AS5 (also dark shaded), taken on days 07 and 42, were used for pyrosequencing.
(TIF)

Figure S2 **Denaturing gradient gel electrophoresis (DGGE) of gut microbiota on day 28.** Each lane displays the banding pattern (fingerprint) of gut microbiota from an individual fish. Samples were taken from different systems on experimental day 28. Ra and Rb: Recirculating aquaculture systems a and b, from tank 1 to 5, AS1-5: Active suspension systems 1 through 5, m: standard reference marker consisting of an amplicon mix of 10 different cloned bacterial 16S rRNA genes used for digital gel normalization.
(TIF)

Figure S3 **Denaturing gradient gel electrophoresis (DGGE) of gut microbiota of system Rb over time.** Each lane displays the banding pattern (fingerprint) of gut microbiota from an individual fish. Samples were taken from the same system on experimental day 07, 28 and 42. Rb: Recirculating aquaculture systems b, from tank 1 to 5, m: standard reference marker consisting of an amplicon mix of 10 different cloned bacterial 16S rRNA genes used for digital gel normalization.
(TIF)

Figure S4 **Denaturing gradient gel electrophoresis (DGGE) of water microbiota from system Rb over time.** Each lane displays the banding pattern (fingerprint) of water microbiota from each tank. Samples were taken from the same system on experimental day 00, 07 and 28. Rb: Recirculating aquaculture systems b, from tank 1 through 5, m: standard reference marker consisting of an amplicon mix of 10 different cloned bacterial 16S rRNA genes used for digital gel normalization.
(TIF)
