# Supplementary figures and images for: The Colonization Dynamics of the Gut Microbiota in Tilapia Larvae
Source: PLoS One. 2014 Jul 29;9(7):e103641. doi: 10.1371/journal.pone.0103641 (PMC4114968; doi:10.1371/journal.pone.0103641)

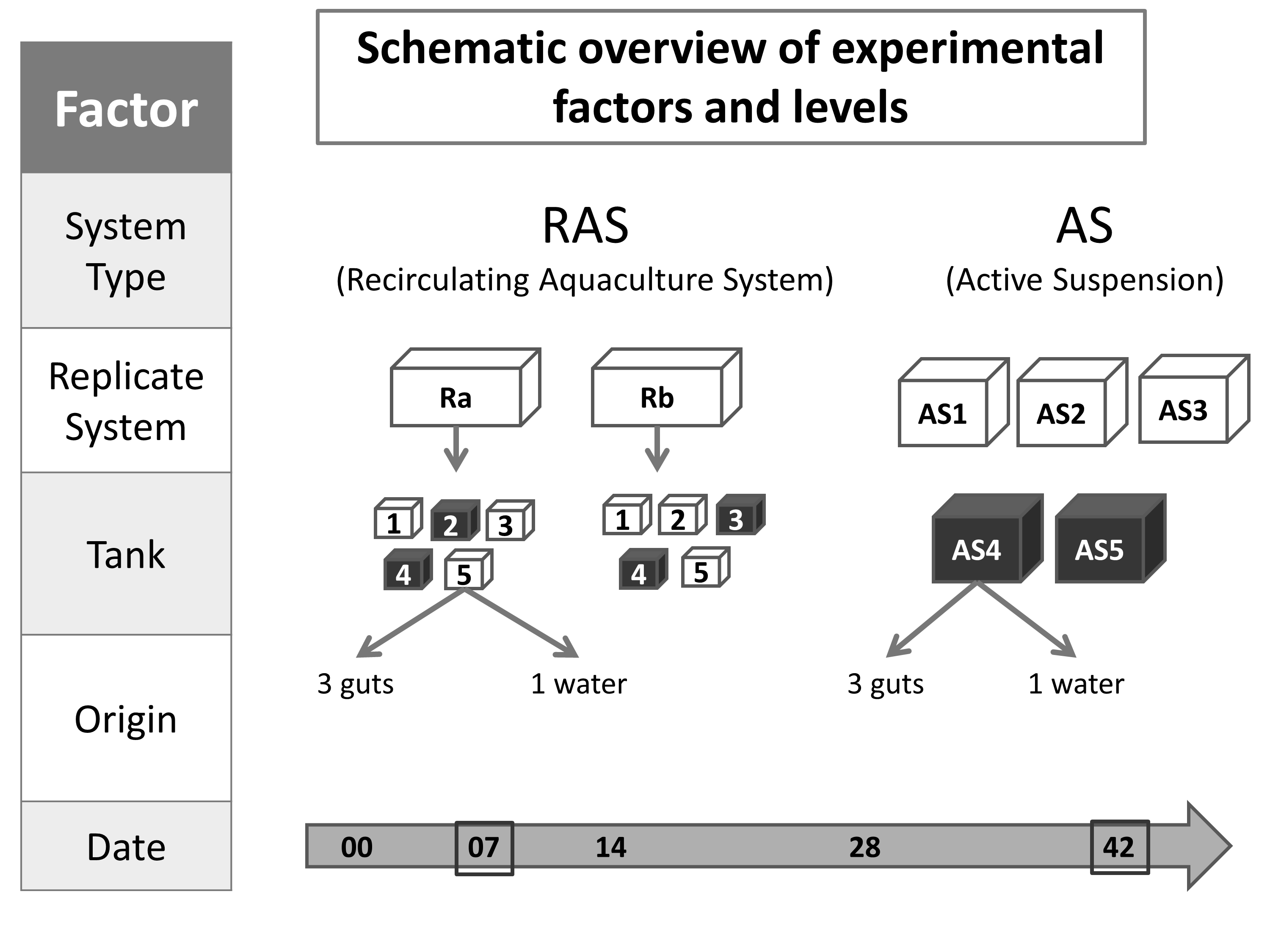

Supplement: Figure S1 — Schematic overview of experimental factors and levels. Five active suspension (AS) and 2 recirculating aquaculture systems (RAS) were used. The replicate RAS are named Ra and Rb; the replicate AS systems are named AS1 through AS5. Each RAS contained five tanks which shared the same water source. AS systems did not have sub-divisions. For DGGE analysis, three guts and water were sampled from each tank in RAS (10 tanks total) and each AS (5 systems) on sampling day 00, 07, 14, 28 & 42. Sub-sets of samples for DGGE of Ra2, Ra4, Rb3 and Rb4 (dark shaded tanks), and active suspension systems AS4 and AS5 (also dark shaded), taken on days 07 and 42, were used for pyrosequencing. (TIF) [file pone.0103641.s001.tif]
